# The Addition of Nature Identical Flavorings Accelerated the Virucidal Effect of Pure Benzoic Acid against African Swine Fever Viral Contamination of Complete Feed

**DOI:** 10.3390/ani11041124

**Published:** 2021-04-14

**Authors:** Hengxiao Zhai, Chihai Ji, Maria Carol Walsh, Jon Bergstrom, Sebastien Potot, Heng Wang

**Affiliations:** 1 DSM (China) Animal Nutrition Research Center, Bazhou 065799, China; 2 Key Laboratory of Comprehensive Prevention and Control for Severe Clinical Animal Diseases, South China Agricultural University, Guangzhou 510642, China; Jichihai@163.com; 3 DSM Nutritional Products, 4303 Kaiseraugst, Switzerland; Maria.Walsh@dsm.com (M.C.W.); Sebastien.Potot@dsm.com (S.P.); 4 DSM Nutritional Products, Parsippany, NJ 07054, USA; Jon.Bergstrom@dsm.com

**Keywords:** African swine fever, benzoic acid, essential oils, virus

## Abstract

**Simple Summary:**

The African swine fever (ASF) virus is one of the deadliest viruses plaguing the global swine industry. There are no effective treatments for pigs becoming infected with the ASF virus. Eliminating infected pigs is the only effective method to tackle an outbreak. Whole farm biosecurity is therefore critical to prevent the virus from entering in the first place. In recent years, feed and feed ingredients have been identified as potential vectors of viral diseases including ASF. The objective of this research was to simulate contamination of feed by ASF virus in a laboratory setting and use this simulation to test the efficacy of some select feed additives for reducing the amount and viability of this virus in feed. The main result shows that the inoculated virus in feed disappeared in a shorter time when the feed had been treated with the compounds. We conclude that these feed additives could be adopted as part of a comprehensive biosecurity system for pig holdings. Pigs might be protected from feed possibly contaminated by this virus, and the pork supply will thus be less likely to be impacted by this viral disease.

**Abstract:**

African swine fever virus is one of the most highly contagious and lethal viruses for the global swine industry. Strengthening biosecurity is the only effective measure for preventing the spread of this viral disease. The virus can be transmitted through contaminated feedstuffs and, therefore, research has been conducted to explore corresponding mitigating measures. The purpose of the current study was to test a combination of pure benzoic acid and a blend of nature identical flavorings for their ability to reduce African swine fever viral survival in feed. This virus was inoculated to feed with or without the supplementation of the test compounds, and the viral presence and load were measured by a hemadsorption test and quantitative real time polymerase chain reaction. The main finding was that the combination of pure benzoic acid and nature identical flavorings could expedite the reduction in both viral load and survival in a swine feed. Therefore, this solution could be adopted as a preventive measure for mitigating the risk of contaminated feed by African swine fever virus.

## 1. Introduction

African swine fever (ASF) is a highly contagious and usually fatal disease for both domestic and feral pigs. This disease has been endemic for decades, initially in sub-Saharan Africa and then in some European regions. In 2018, China, a country holding half of the global population of pigs, reported its first outbreak of ASF, and it quickly spread to some other Asia-Pacific countries. According to the recent update by the World Organization for Animal Health (OIE) [1], approximately 7 million pigs were lost based on notified outbreaks in Asia and the Pacific since 2018. The actual losses could be much worse because of the existence of unreported cases due to farmers’ concerns for potential social and economic consequences in association with reporting disease outbreaks [2], especially when a financial compensation program does not exist or seem equitable. On the country level, trade embargos for pork are frequently imposed when this virus is found to be present, as exemplified by the recent Asian market ban on German pork imports after their first confirmed case of ASF in 2020. Embargos can be devastating for the pig farming industry in the affected countries, and disruptive for the balance between supply and demand of pork. Therefore, it is of paramount importance to safeguard the ASF-free countries against entry of the virus.

The African swine fever virus is a large, enveloped virus with an average diameter of 200 nm and a genomic core consisting of a single molecule of linear, double-stranded deoxyribonucleic acid (DNA) [3]. This virus has been determined to be extremely resistant to harsh physical conditions such as high temperatures, putrefaction and desiccation, freezing/thawing, and extreme pH values [4]. Low temperatures and the presence of blood, feces and tissues are favorable for longer survival times. This inherent high resistance and stability endows this virus with diverse transmission routes and thereby renders it extremely difficult to eradicate. The contact of infected pigs via infectious bodily fluids, as well as by aerosol over short distances between pens, has been demonstrated to be the main route of ASF transmission among pigs [5]. After entering the pig’s body via the pharyngeal tonsil, which is the most common route of entry [6], the pathogenesis of ASF is characterized by viral replication in cells of the monocyte/macrophage lineage, depletion of lymphoid tissues, and impairment of hemostasis and immune functions [7]. The massive destruction of naïve lymphocytes by cytokines released by infected lymphocytes, and not the virus per se, plays a major role in the development of ASF [7,8]. Since there are still not any effective vaccine or curative treatments for ASF, biosecurity and culling of infected pigs are the only effective methods to fight the virus [9,10,11].

An effective biosecurity program entails a comprehensive approach that addresses every potential threat enabling viral entry into pig holdings. According to a recent review by Jones et al. [12], ASF virus warrants a moderate risk characterization for transmission through the American feed supply chain, considering both its severity and probability. As a fact stated by the OIE [13], ASF virus can be spread by contaminated feed due to its high environmental resistance. In China, feed ingredients contaminated by ASF virus were identified as a very likely source for the spread of ASF, and inspection of pig feed was thus advocated as a preventative measure [14]. The transmission of virus is determined first by the likelihood of viral contamination, secondly, by the survivability of the virus, and finally, by the infectivity of the remaining virus in the vector [12]. The African swine fever virus Georgia 2007 survived in multiple feed ingredients with a relatively consistent half-life (4.1 to 5.1 days) similar to that of the stock virus, based on limited data [15,16]. With an updated modeling approach, the virus half-life was extended to 14.2 days in complete feed, and the feed matrix was recognized to be capable of prolonging the survival of ASF virus [17]. Moreover, it has been demonstrated that ASF virus can be easily transmitted orally through the natural consumption of feed, and this likelihood of infection could escalate by repeated exposures [18].

As part of a comprehensive biosecurity program, feed biosecurity should be included. Proactive mitigation through quarantining ingredients, thermal processing, or the use of feed additives as a final barrier should be considered. Additionally, feed additives may be more successful in terms of preventing cross-contamination [12]. The traditional use of pure benzoic acid for preserving food and beverages against fungi and bacteria, along with the well-documented antiviral effects of nature identical flavorings in vitro, is suggestive for the possibility that these compounds could mitigate the transmission of viruses in feed. Gebhardt et al. [19] demonstrated that the combination of pure benzoic acid and nature identical flavoring enhanced the degradation of ribonucleic acid (RNA) of porcine epidemic diarrhea (PED) virus in feed. Dee et al. [20] devised an ice-block model to mimic the contamination of feed by porcine reproductive and respiratory syndrome (PRRS) virus, Seneca virus A (SVA), and PED virus, and demonstrated that the pigs fed diets including the combination of pure benzoic acid and nature identical flavorings showed improved health and performance as compared to the pigs from the positive control viral infected group. However, ASF virus was not tested in these studies, and the protective effects of this combination could not be assumed for ASF virus considering ASF virus is a DNA virus and renowned for its stability in the environment.

Therefore, the aim of this study was to simulate the contamination of feed with ASF virus in a laboratory setting and validate whether the combination of pure benzoic acid and nature identical flavorings could exert similar effects on the viability and load of ASF virus as previously reported for other viruses. This study was based on the hypothesis that the addition of nature identical flavorings to pure benzoic acid could decrease both the viral load and viability in complete feed when contamination of ASF virus occurred.

## 2. Materials and Methods

### 2.1. General

The test products were either pure benzoic acid (99.9% benzoic acid; DSM (China) Limited, Shanghai, China) or the combination of pure benzoic acid and a mixture of nature identical flavorings including thymol, eugenol, piperine, and curcumin as the main ingredients (DSM Nutritional Products, Grenzach, Germany; Table 1). Pure benzoic acid and the nature identical flavorings were mixed at the ratio of 10:0.4 by weight which was established by referring to the effective levels of 5 g/kg of pure benzoic acid and 0.2 g/kg of the nature identical flavorings when used separately in feed for pigs [21].

There are 4 treatment groups: a negative control without the inoculation of ASF virus (NC), a positive control with the inoculation of ASF virus (PC), and the NC with the inoculation of ASF virus and supplemented with either 0.5% pure benzoic acid or the combination of 0.5% pure benzoic acid and 0.02% nature identical flavorings. The assays were performed in 2 matrices, i.e., having the test products either dissolved in phosphate-buffered saline (PBS) or mixed in a pig diet. The assays in PBS were considered secondary and supportive for assays in feed matrix considering the poor solubility of pure benzoic acid and nature identical flavorings in water. All the operations with the ASF virus were carried out in a biosafety level (BSL)-3 laboratory at South China Agricultural University (Guangzhou, China).

### 2.2. Cytotoxicity Assays

The supernatant of the treatments in feed matrix and aliquots of the treatments in PBS were tested for their cytotoxicity according to the cell counting kit-8 (CCK-8, Takara, Japan) assay. Briefly, 100 µL of cell suspension were dispensed in a 96-well plate and incubated for 3 h in an incubator (37 °C, 5% CO_2_); a series of dilutions (0-, 10-, 100-, and 1000-fold) of the supernatant or the aliquot were added to the wells and incubated for 3 h; 10 µL of CCK-8 solution was added to each well of the plate and incubated for 1 h; and finally, absorbance at 450 nm was measured using a microplate reader (Multiskan FC, Thermo Fisher Scientific, Salt Lake City, UT, USA). Each dilution was tested in 3 replicates.

### 2.3. Assay in Phosphate-Buffered Saline

Four treatment solutions were prepared with PBS according to the previously defined 4 treatments. Four 20-mL of the solution of PC or with test compounds were pipetted into 4 centrifuge tubes (50 mL, Thermo Fisher Scientific, Massachusetts, MA, USA), then inoculated with 1 mL ASF virus with the titer of 10^5.3^ 50% hemadsorption doses (HAD_50_)/mL (ASFV/China/GZ201801), whereas 1 mL cell culture solution was added to the 20 mL solution of NC. The 4 test tubes of each treatment group corresponded to day 1, 3, 6, and 9 post inoculation as the sampling days. The test tubes were continuously agitated at 1200 rpm/min. On each of the pre-determined sampling days, one tube per treatment was taken for analysis. Aliquots of the solutions (100 µL) after 10-fold dilution were used to infect porcine alveolar macrophages in plate wells for 2 h before the removal of the supernatant and then the addition of RPMI 1640 medium (Gibco, Thermo Fisher Scientific, Salt Lake City, UT, USA). After further 24-h incubation, the real-time quantitative polymerase chain reaction (RT-PCR) and hemadsorption test were performed. The primary alveolar macrophages were collected from piglets tested negative for PRRS virus, porcine circovirus 2, and pseudorabies virus.

### 2.4. Assay in Feed Matrix

Four samples of commercial pig feed with corn and soybean meal as the main ingredients were mixed individually in a miniature blade mixer according to the previously defined treatments. These samples were gamma-irritated at 25 kGy. Four 10-g sub-samples of each treatment were weighed into 4 centrifuge tubes (50 mL, Thermo Fisher Scientific, USA) to be sampled on day 1, 3, 6, and 9 post inoculation, respectively. Each sample tube of the PC and the treatments with supplements was inoculated with 1 mL 10^5.3^ HAD_50_/mL ASF virus (ASFV/China/GZ201801), whereas 1 mL cell culture solution was added to the tubes of NC group. The sample tubes were continuously agitated at 1200 rpm/min to prevent clumping. On each of the pre-determined sampling days, one sample tube per treatment was taken for analysis. Ten mL sterile PBS were added to each sample tube for collection of supernatant after centrifugation with aseptic techniques. One aliquot (100 µL) of the supernatant after 100-fold dilution was used to infect porcine alveolar macrophages in plate wells for 2 h before the removal of supernatant and then the addition of RPMI 1640 medium. After a further 24-h incubation, RT-PCR and hemadsorption test were performed.

### 2.5. Hemadsorption and Real-Time PCR Analysis

For hemadsorption, 20 µL 1% red blood cells were added to each well for overnight incubation. On the following day, the agglutinated red blood cells with a ring appearance were observed under a microscope [22].

The DNA was extracted with a commercial Axygen kit (Corning, Shanghai, China) and then stored at −20 °C before analysis by RT-PCR with a commercial kit for detecting ASF virus (Beijing MingRiDa Technology Co. Ltd., Beijing, China) according to the manufacturer’s instructions. This assay targets the VP72 gene of the ASF virus and uses the 5′-nuclease assay system to detect the amplicons. The analytical results were presented as cycle threshold (Ct) at which virus was detected. A greater Ct value indicates less genetic materials in the test samples. Samples were declared negative when Ct value is greater than 40 and positive when less than 38.

### 2.6. Statistical Anaysis

Data were analyzed with Fit model platform of JMP (15.1.0; 2019 SAS Institute Inc., Cary, CN, USA) to determine the main effects of sampling time, treatment, and their interaction. Tukey’s test were used to compare the means. The least square means were presented. Significance was declared at *p* < 0.05.

## 3. Results

### 3.1. Cytotoxcicity Assays

The survival rate of cells in the presence of 100- and 1000-fold dilutions of the supernatant of feed treated with pure benzoic acid or pure benzoic acid combined with nature identical flavorings reached 100% and thereby, 100-fold dilution was used in downstream hemadsorption tests to ensure no toxic effects on the porcine alveolar macrophage cells (Table 2). With the test compounds in PBS solution, 10-fold dilution was chosen for the hemadsorption tests because this dilution already resulted in a cell survival rate of 99%.

### 3.2. Assay in Phosphate-Buffered Saline

The NC samples tested negative for hemadsorption and were confirmed to be free of ASF virus DNA by RT-PCR on all sampling days, while the PC samples were all positive for hemadsorption and had an initial Ct value of 28.4 on day 1 post inoculation (Table 3). This Ct value decreased with time and reached a significant lower value of 27.1 on day 9 post inoculation, indicating a paradoxical increase in DNA of ASF virus. The inclusion of pure benzoic acid or its combination with nature identical flavorings resulted in 2 positive hemadsorption tests on day 6 post inoculation and all negative tests on day 9 post inoculation. In keeping with the general decreasing number of positive tests with time, the Ct values increased gradually in the presence of either pure benzoic acid or its combination with nature identical flavorings indicating a decrease in DNA of ASF virus or an increase in its degradation. On day 9 post inoculation, the combinational treatment led to a Ct value of 40.5 indicating a change of the sample from being positive to negative. In addition, the Ct values were significantly higher for the supplemented treatments than the PC at all sampling days.

### 3.3. Assay in Feed Matrix

The NC samples tested negative for hemadsorption and were confirmed to be free of ASF virus DNA by RT-PCR on all sampling days, while the PC samples were all positive and had an initial Ct value of 30.4 on day 1 post inoculation and then remained relatively stable from day 3 to 9 post inoculation (Table 4). The inclusion of pure benzoic acid resulted in a decrease from 4 positive hemadsorption tests on day 1 to 3 on day 3 and then complete negativity on day 6 and 9 post inoculation. Complete negative tests were achieved already on day 1 post inoculation with the combinational treatment. In general, the Ct values increased gradually in the presence of either pure benzoic acid or its combination with nature identical flavorings and the majority were significantly higher than the PC. Additionally on day 9 post inoculation, the Ct values exceeded 40 as the cut-off value for positivity as interpreted as a change of the samples from a positive status to negative.

## 4. Discussion

Traditionally, both the pure benzoic acid and the nature identical flavorings evaluated in this study are supplemented to feed for improving the growth and health performance of pigs. Pure benzoic acid is a common preservative for foods and beverages and is an efficacious acidifier for weaned piglets and fattening pigs [23,24]. Pure benzoic acid can improve growth performance of pigs via multifaceted mechanisms such as selective suppression of the pathogens in the gut, enhancement in absorptive and antioxidative capacity of gut, and improvement in biodiversity in gastro-intestinal microbiota [25,26,27,28,29]. The use of nature identical flavorings in combination with pure benzoic acid has been shown to reduce the amount of pure benzoic acid needed for maintaining the same performance of pigs supplemented with greater levels of pure benzoic acid alone [21], indicating that there is a synergistic effect when combining pure benzoic acid and nature identical flavorings. This synergism is plausible, given the acidifying effect of pure benzoic acid in contrast to nature identical flavorings that are hydrophobic and capable of disrupting the cell membrane of some pathogens [30]. Recently, this combination has been demonstrated as an alternative to the use of antibiotics for pigs challenged with *Escherichia coli* F4 [31].

The application of pure benzoic acid and nature identical flavorings for the control of viral transmission through feed is still novel, despite the broad recognition that feed is a valid vector for transmitting viruses. The first evidence that contaminated feed can serve as a vehicle for PED virus infection of naïve pigs was provided by Dee et al. [32], with contaminated feed collected from bins for 3 clinically affected breeding herds. Later, by dropping an ice block carrying equal concentrations of PRRS virus, SVA, and PED virus into feed bins, Dee et al. [20] demonstrated that these viruses entered the pig room via feed and the conveyance of feed into the barn, where the pigs in the room consumed the feed and were exposed to the viruses, which resulted in the pigs becoming infected. In Dee et al.’s study, the combination of pure benzoic acid and nature identical flavorings at different inclusion rates was found to improve pig health and performance when these viruses were placed in the feed when compared to the non-supplemented pigs. This seminal study prompted us to explore the possibility for mitigating effects of pure benzoic acid and nature identical flavorings against the transmission of ASF virus through feed, which is of great relevance to regions where ASF is present or there is considerable risk for its introduction [33].

This study has demonstrated two components of significance. Firstly, the ASF virus was successfully inoculated both to the PBS solution and a complete feed and survived for 9 days (the longest duration tested in this study) as shown by the PC in comparison to the NC. These procedures could be used as models for evaluating the efficacy of other potential compounds for mitigating ASF viral transmission in similar vectors. Secondly, both pure benzoic acid and its combination with nature identical flavorings showed efficacy for impairing the viability of live ASF virus and decreasing the viral load in the matrices, which provided supportive evidence for the hypothesis of the current study. The potential mechanisms could be related to the viral envelop being degraded by the acid, since its integrity is indispensable for accomplishing the cellular infection [34], while the nature identical flavorings exert antiviral activity via integration with the envelop [35].

The results of the current study imply that both water and feed could harbor the ASF virus and then infect pigs, which calls attention to the need for mitigation strategies as part of a comprehensive biosecurity program for pig holdings. Feed might pose a greater threat in comparison to water sources because feed delivery is a high-frequency event and the likelihood of infection via contaminated feed increases dramatically with increasing exposures [18]. The application of the combinational compounds as a preventive practice for safeguarding the biosecurity of feed appears promising. The combination of pure benzoic acid and nature identical flavorings appeared to have a stronger efficacy against live ASF virus than the pure benzoic acid alone in a feed matrix according to the hemadsorption test in the current study, whereas PBS medium did not differentiate the antiviral effects between pure benzoic acid alone and the mixture.

We acknowledge some limitations with this study. The inoculated dose of ASF virus might not represent all situations in reality. The mitigating effects of the test compounds were not confirmed with a bioassay study with the mitigated or non-mitigated vectors fed to live pigs. Further study is warranted to understand the mechanism by which adding nature identical flavorings to pure benzoic acid seems to provide a boosting effect for reducing the survival of virus in feed matrix. It is also worth emphasizing that mitigating the risk of viral spread via feed with feed additives can be an important component of a holistic biosecurity system, but does not reduce the importance of other preventative biosecurity practices.

## 5. Conclusions

In conclusion, the current study developed laboratory models simulating the contamination of PBS and a complete swine feed with ASF virus and validated the mitigating effects of some feed additives. Under the conditions of this study, it was demonstrated that both pure benzoic acid and its mixture with nature identical flavorings can reduce the viral load and survivability of the ASF virus in these matrices, and the combinational solution appeared to be more advantageous when compared to pure benzoic acid alone in terms of preventing the survival of ASF virus in feed faster.

## Figures and Tables

**Table 1:** Test compounds.

Type	Organic Acid	Nature Identical Flavorings
Compounds	Pure benzoic acid	Thymol, eugenol, piperine, curcumin

**Table 2:** Survival rate of cells in the presence of different dilutions of either the supernatant of feed or the phosphate-buffered saline (PBS) solution treated with pure benzoic acid or its combination with nature identical flavorings (%).

**Dilution**	**Feed**	
	**Pure Benzoic Acid**	**Pure Benzoic Acid and Nature Identical Flavorings**
No dilution	68.8	45
10-fold dilution	85	88.5
100-fold dilution	100	100
1000-fold dilution	100	100
**Dilution**	**PBS Solution**	
	**Pure Benzoic Acid**	**Pure Benzoic Acid and Nature Identical Flavorings**
No dilution	90	95
10-fold dilution	99	99
100-fold dilution	100	100
1000-fold dilution	100	100

**Table 3:** Effect of pure benzoic acid and the combination of pure benzoic acid and nature identical flavorings on hemadsorption and detection of ASF virus by real time-PCR at different days post inoculation (dpi) in phosphate-buffered saline.

**Treatment**	**Hemadsorption ^1^**			
	**1 dpi**	**3 dpi**	**6 dpi**	**9 dpi**
Negative control	− − − −	− − − −	− − − −	− − − −
Positive control	+ + + +	+ + + +	+ + + +	+ + + +
Pure benzoic acid	+ + − −	+ − − −	+ + − −	− − − −
Pure benzoic acid and nature identical flavorings	+ + + +	+ + − −	+ + − −	− − − −
**Treatment**	**PCR Results ^2^**			
	**1 dpi**	**3 dpi**	**6 dpi**	**9 dpi**
Negative control	0	0	0	0
Positive control	28.4 ^f^	28.0 ^fg^	28.1 ^fg^	27.1 ^g^
Pure benzoic acid	33.5 ^e^	35.7 ^d^	37.6 ^c^	38.8 ^b^
Pure benzoic acid and nature identical flavorings	33.0 ^e^	32.7 ^e^	39.4 ^ab^	40.5^a^

^1^ (−) indicates no rosette formation and (+) indicates hemadsoprtion; ^2^ Main effect of day on Ct value, *n* = 9, SEM = 0.13, *p* < 0.01; main effect of treatment on Ct value, *n* = 12, SEM = 0.11, *p* < 0.01; treatment × day interaction effect on Ct value, *n* = 3, SEM = 0.22, *p* < 0.01; ^a,b,c,d,e,f,g^ Means within interaction lacking a common superscript differ (*p* < 0.05); dpi, days post inoculation.

**Table 4:** Effect of pure benzoic acid and the combination of pure benzoic acid and nature identical flavorings on hemadsorption and detection of African swine fever (ASF) virus by real-time PCR at different days post inoculation (dpi) in the feed matrix.

**Treatment**	**Hemadsorption ^1^**			
	**1 dpi**	**3 dpi**	**6 dpi**	**9 dpi**
Negative control	− − − −	− − − −	− − − −	− − − −
Positive control	+ + + +	+ + + +	+ + + +	+ + + +
Pure benzoic acid	+ + + +	+ + + -	− − − −	− − − −
Pure benzoic acid and nature identical flavorings	− − − −	− − − −	− − − −	− − − −
**Treatment**	**PCR results ^2^**			
	**1 dpi**	**3 dpi**	**6 dpi**	**9 dpi**
Negative control	0	0	0	0
Positive control	30.4 ^e^	34.3 ^d^	34.9 ^d^	34.5 ^d^
Pure benzoic acid	35.6 ^cd^	34.8 ^d^	39.4 ^ab^	40.5 ^a^
Pure benzoic acid and nature identical flavorings	35.2 ^cd^	38.2 ^b^	37.3 ^bc^	40.6 ^a^

^1^ (−) indicates no rosette formation and (+) indicates hemadsoprtion; ^2^ Main effect of day on Ct value, *n* = 9, SEM = 0.26, *p* < 0.01; main effect of treatment on Ct value, *n* = 12, SEM = 0.22, *p* < 0.01; treatment × day interaction effect on Ct value, *n* = 3, SEM = 0.44, *p* < 0.01; ^a,b,c,d,e^ Means within interaction lacking a common superscript differ (*p* < 0.05).

## Data Availability

The data presented in this study are available on request from the corresponding author. The data are not publicly available due to privacy protection.
